# Evolution of Electrocardiographic Repolarization Parameters During Antiandrogen Therapy in Patients with Prostate Cancer and Hypogonadism

**DOI:** 10.1007/s12012-020-09566-6

**Published:** 2020-03-09

**Authors:** Andrei Cristian Dan Gheorghe, Ana Ciobanu, Andreea Simona Hodorogea, George Daniel Radavoi, Viorel Jinga, Ioan Tiberiu Nanea, Gabriela Silvia Gheorghe

**Affiliations:** 1 Carol Davila University of Medicine and Pharmacy, Bucharest, Romania; 2 Department of Internal Medicine and Cardiology, Theodor Burghele Clinical Hospital, Bucharest, Romania; 3 Department of Urology, Theodor Burghele Clinical Hospital, Bucharest, Romania

**Keywords:** QT interval, Tpeak-tend wave interval, Hypogonadism, Enzalutamide, Degarelix

## Abstract

We assessed the effects of antiandrogen therapy on ECG parameters of ventricular repolarization related to arrhythmic risk in 35 patients aged 70.3 ± 7 years with advanced prostate cancer treated with degarelix associated with enzalutamide (group A, 26 patients) or degarelix monotherapy (group B, 9 patients). We analyzed Fridericia corrected Q-T interval (QTc), Q-T dispersion (QTd), J-Tpeak interval (JTp), mean and maximum Tpeak-Tend interval (Tpe) and Tpe/QT ratio, Tpeak-Tend dispersion (Tped), index of cardio-electrophysiological balance (iCEB) from ECG tracings, and occurrence of ventricular premature beats (VPB) recorded by Holter ECG, before initiation of medication (M0) and after 6 months of treatment (M1). The groups had similar demographics except for a higher prevalence of prior myocardial infarction in group B (*p* = 0.01). All patients had low serum testosterone at M1. Baseline QTc, QTd, maxTpe/QT, meanTpe, maxTpe, Tped values were higher in B compared to A. They had a significant prolongation at M1 only in A. 20 patients in A and 6 in B had a 10% prolongation or decrease of iCEB (*p* = 0.66). In 5 patients, VPB severity increased from non-complex to complex: 3 in A and 2 in B (*p* = 0.31), but no sustained ventricular arrhythmia was registered. In conclusion, after 6 months of treatment, patients with hypogonadism on degarelix associated with enzalutamide had significant prolongation of QTc, QTd, maxTpe, meanTpe/QT, maxTpe/QT, Tped compared to patients on degarelix alone. The proportion of patients with 10% iCEB variation was similar between groups. There was no record of severe arrhythmias during the first 6 months of treatment.

## Introduction

Chemotherapy, targeted therapy, and monoclonal antibodies therapy used in oncological and autoimmune systemic diseases have many cardiovascular deleterious effects according to the cardio-oncology guidelines of the European Society of Cardiology [1], but there are also concerns regarding the cardiac effects of hormone therapy. Prostate cancer is the third cause of cancer in men over 60 years [2]. Patients with advanced prostate cancer receive androgen deprivation therapy inducing hypogonadism [2], which is known to damage the cardiac repolarization parameters and to increase the risk of torsade de pointes or other severe ventricular arrhythmias [3, 4]. At the same time, there are also data suggesting that drugs used to induce hypogonadism, such as gonadotropin-releasing hormone agonists, gonadotropin-releasing hormone antagonists, cytochrome-17 inhibitors, nonsteroidal androgen receptor antagonists and 5α-reductase inhibitors, prolong QTc interval and increase the risk of torsade de pointes [5]. However, these data are controversial and, for example, the website crediblemeds.org currently lists only gonadotropin-releasing hormone antagonist degarelix and gonadotropin-releasing hormone agonist leuprolide as possible risks for torsade de pointes [5].

Several electrocardiographic indices are recommended for the assessment of cardiac repolarization and arrhythmic risk stratification: corrected Q-T interval (QTc), QT interval dispersion (QTd), Tpeak—Tend interval (Tpe), Tpeak—Tend/QT ratio (Tpe/QT), Tpeak—Tend interval dispersion (Tped), J-Tpeak interval (JTp). Unfortunately, none of them is an ideal parameter for the assessment of the arrhythmic risk [6]. Prolongation of QTc is associated with prolongation of action potential duration at cellular level, which is involved in a higher risk of occurrence of early afterdepolarization, and triggers activity responsible for arrhythmia [7]. In patients receiving chemotherapy, the prolongation of QTc of more than 60 ms increases the risk of cardiac arrhythmia [1]. However, the measurement of QTc has low sensibility and specificity and does not correlate with the heterogeneities of myocardial repolarization [7]. QTd prolongation increases the arrhythmic risk, but it is not a reliable predictor of arrhythmogenesis [7]. Tpe is related to the global dispersion of repolarization and the arrhythmic risk in some but not all forms of congenital long QT syndrome, in ischemic heart disease and hypertension [8]. The problem is its inter-individual variability and dependence on the heart rate. Tpe/QT is constant and seems more useful in predicting arrhythmic risk [7]. Additional repolarization indices, such as JTp and Tpe/JTp, do not appear to be superior to the aforementioned tests [7]. However, the concomitant prolongation of JTp and QTc intervals is associated with an increased risk of cardiac arrhythmias [7]. The index of cardio-electrophysiological balance (iCEB) is proposed for the assessment of the cardiac repolarization and conduction [7]. The prolongation or decrease of iCEB more than 10% from the baseline values seems sensitive to the cardiac repolarization changes induced by drugs [7, 9].

We aimed to assess the effects of enzalutamide in co-treatment with degarelix (group A) versus degarelix in monotherapy (group B), on ECG parameters of ventricular repolarization related to arrhythmic risk in patients with advanced prostate cancer and induced hypogonadism.

## Methods

A longitudinal observational analytical study was conducted and included consecutive patients with the diagnosis of advanced prostate cancer in whom treatment with degarelix associated or not with enzalutamide was indicated by the urological and oncological team. The study was conducted according to the ethical principles stated in the Declaration of Helsinki. The study protocol has been approved by the local ethics committee. Patients were informed about the aims of the study and all of them signed the informed consent.

The screening of each patient was made 5–7 days before the initiation of androgen deprivation therapy. Patients were considered eligible if they were in sinus rhythm and if they had no cardiac diseases or had at most stable coronary disease, treated arterial hypertension, myocardial infarction more than 6 months before screening (old myocardial infarction, OMI), heart failure NYHA class I-II, left ventricular ejection fraction ≥ 45%, estimated glomerular filtration rate > 30 mL/kg/min assessed with MDRD formula, diabetes mellitus with glycosylated hemoglobin ≤ 7.5%, normal serum potassium, magnesium, and calcium levels.

Exclusion criteria were unstable angina, myocardial infarction during 6 months before screening, heart failure NYHA class III–IV, left ventricular ejection fraction < 45%, sustained ventricular tachycardia, persistent or permanent atrial fibrillation, complete left bundle branch block, complete right bundle branch block, diabetes mellitus with glycosylated hemoglobin > 7.5%, chronic kidney disease grade 4–5, chronic use of drugs known to prolong QTc, life expectancy less than 6 months.

Patients were distributed into two treatment groups based on the urological and oncological recommendations: degarelix in association with enzalutamide (group A) and degarelix in monotherapy (group B). The dose of enzalutamide was 160 mg per os, once daily, and the initial dose of degarelix was 240 mg followed by 80 mg every 28 days, given subcutaneously.

All patients had a clinical examination, laboratory tests, ECG, Holter ECG, and measurement of left ventricular ejection fraction by echocardiography, before the initiation of medication (M0) and after 6 months of treatment (M1).

No patient received drugs that prolong QT interval during the 6-month follow-up period.

Ventricular repolarization parameters were measured using resting 12-lead ECG on three different consecutive complexes for each lead and their mean values were calculated. The ECG leads were considered uninterpretable if the T-wave amplitude was lower than 0.1 mV or if biphasic T-waves were present. The measurements were performed on a stable RR interval, with a heart rate between 50 and 90 beats/min [6].

**Fig. 1:**
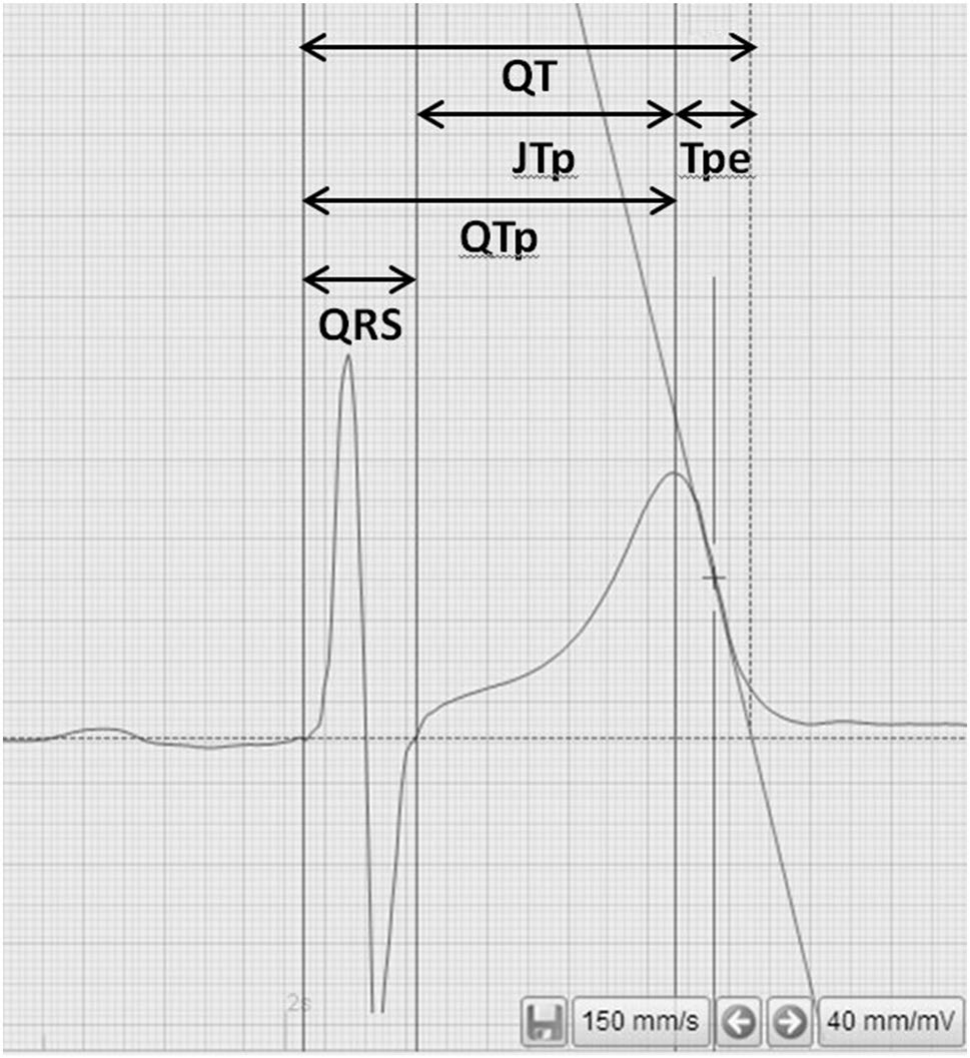
Example of measurement of ECG repolarization parameters

QT interval, defined as the interval between the onset of the QRS complex and the end of the T wave, was measured in all leads. Its maximum value was then used. The end of the T wave was determined by the method of the tangent to the steepest slope of the descending portion of the T wave (Fig. 1). QTd was calculated as the difference between the maximum and minimum QT intervals.

The following parameters were measured only in precordial leads and their mean values were then calculated:

- Tpeak–Tend interval (Tpe), defined as the interval between T wave peak and T wave end; its maximum value was also used;
- Q-Tpeak interval (QTp)—the interval between the onset of the QRS complex and the peak of T wave;
- J-Tpeak (JTp)—the interval between J point and the peak of T wave;
- J-Tend (JTe)—the interval between J point and the end of T wave;
- index of cardio-electrophysiological balance (iCEB) was calculated as QTc interval divided by QRS duration; we noted maximum iCEB (max iCEB) and calculated mean iCEB (mean iCEB) [9].

Tpe interval dispersion (Tped) was defined as the difference between the highest and the lowest value of Tpe intervals.

QT, QTd, QTp, JTp, Tpe, and Tped were corrected for heart rate using Fridericia formula (QTc = QT/$\sqrt[3]{}$ RR) that demonstrated the best prediction for short- and long-term mortality [10]. We also calculated mean Tpe/QT ratio and maximum Tpe/QT ratio.

We defined the amount of variation “delta” for the ECG parameters as the difference between the values at M1 minus the value at M0.

We used Common Terminology Criteria for Adverse Events (CTCAE) version 5 for the classification of the severity of QTc interval prolongation in 4 grades: grade 1—QTc duration 450–480 ms; grade 2—QTc duration 481–500 ms; grade 3—QTc duration > 500 ms or > 60 ms change from baseline; grade 4—torsade de pointes; polymorphic ventricular tachycardia; signs or symptoms of serious arrhythmia [11].

An iCEB variation of more than 10% (either an increase or a decrease) was considered significant.

We also measured QRS complex and PR interval durations.

The method used for assessment of ECG parameters has been previously tested in another study performed by members of this team, estimating the inter-observer variability of the measurements by the intra-class correlation coefficient using an absolute agreement definition. The results showed an excellent agreement between the observers, with coefficients over 0.9 [6].

24-h Holter ECG monitoring was used to assess the number and severity of the VPBs, after manually excluding noise, artifacts, atrial ectopy with aberrancy. Complex VPBs were defined as occurrence of bigeminy, trigeminy, couplets, or unsustained ventricular tachycardia during the 24-h monitoring.

For each patient, we compared the variations of ECG repolarization parameters between visits and also the changes in the severity of VPBs.

Data are presented as means ± standard deviation for numerical variables and as absolute numbers and percentages for categorical variables. For numerical variables, parametric (Student’s *t* test for dependent samples or for groups) or non-parametric (Mann–Whitney) tests were used, according to the distribution of data. Also, Levene’s test was used for assessment of the homogeneity of variances. Chi-square test and Fisher’s exact test were used to compare categorical variables. The statistical analysis and the figures were performed using STATISTICA version 8. A *p* value < 0.05 was considered statistically significant.

## Results

**Fig. 2:**
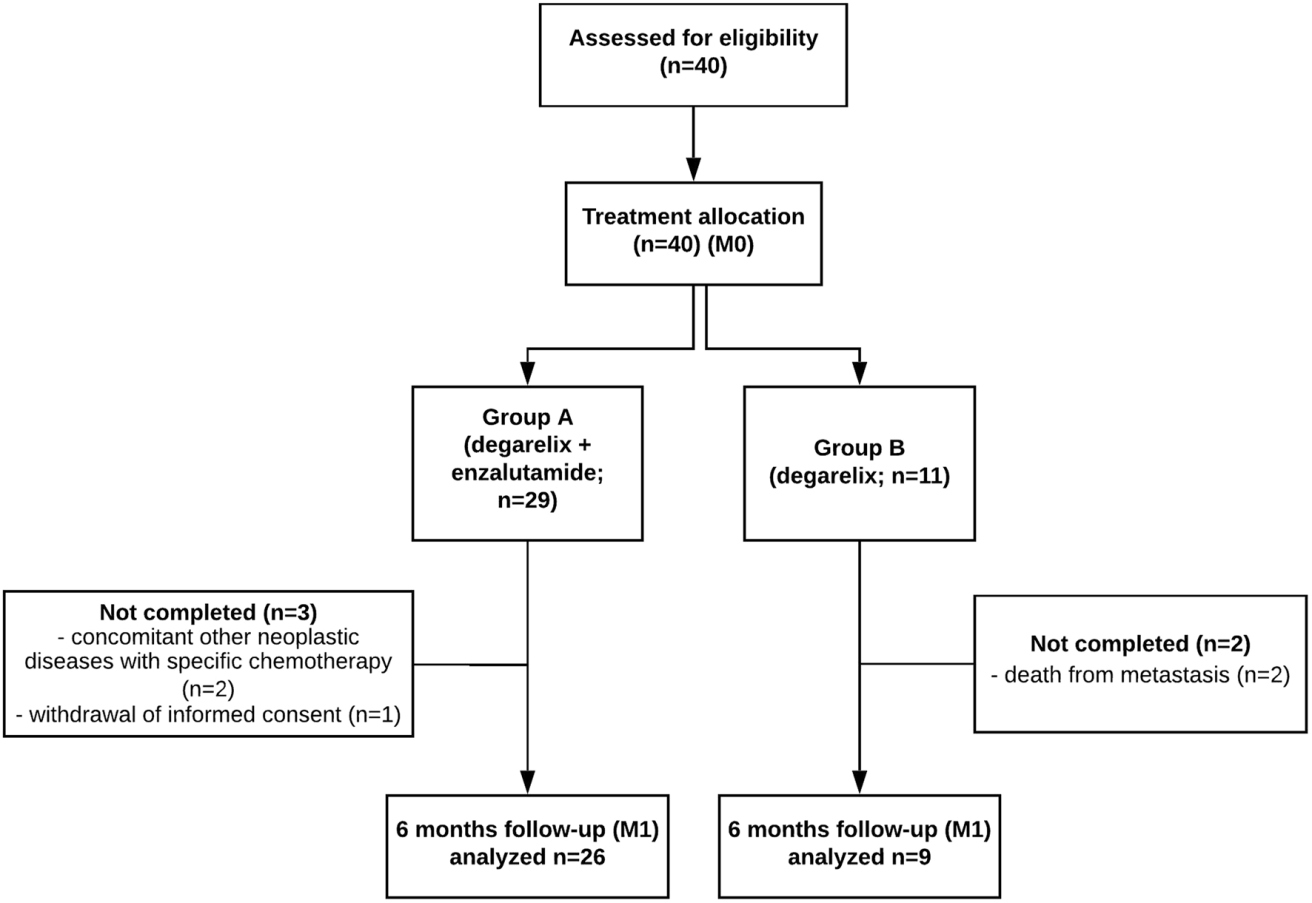
Patient flowchart

A total of 40 patients were included and 35 completed the study. The reasons for the 5 patients leaving the trial after treatment allocation were: other concomitant neoplastic conditions diagnosed in 2 patients who started chemotherapy (Hodgkin disease and gastric neoplasia); death between M0 and M1 in 2 patients due to hepatic and bone metastasis; withdrawal of informed consent in 1 patient. The remaining 35 patients performed the ECG study and 25 of them performed both ECG and Holter studies. 26 (74.3%) patients were in A and 9 (25.7%) in B. Patient flowchart is presented in Fig. 2. The basic demographic characteristics and baseline cardiovascular therapy of the study groups are shown in Table 1. There were no demographic differences between patients in A and B, except for a statistically significant higher prevalence of OMI in B: 14.2% in A versus 44.4% in B, *p* = 0.01.

**Table 1:** Demographic data and basic cardiovascular treatment in the study groups A and B

	Total (*n* = 35)	Group A (*n* = 26; 72.3%)	Group B (*n* = 9; 25.7%)	*p*
Age (years)	70.2 ± 7.0	70.3 ± 7.4	69.8 ± 6.2	0.87
Cardiovascular risk factors and comorbidities				
Body mass index > 27 kg/m^2^	10 (28.5%)	8 (30.7%)	2 (22.2%)	1
Diabetes mellitus	9 (25.7%)	7 (26.9%)	2 (22.2%)	1
Grade 3 chronic kidney disease	5 (14.7%)	4 (15.3%)	1 (11.1%)	1
Hypertension	27 (77.1%)	20 (76.9%)	7 (77.7%)	1
Stable coronary artery disease	19 (54.2%)	13 (50%)	6 (66.6%)	0.46
Old myocardial infarction	5 (14.2%)	1 (3.85%)	4 (44.4%)	0.01
Heart failure NYHA class II	4 (11.4%)	3 (11.5%)	1 (11.1%)	0.97
Left ventricular ejection fraction (%)	60.4 ± 5.0	60.3 ± 4.7	60.9 ± 6.3	0.78
Cardiovascular drugs				
Beta-blockers	19 (54.2%)	12 (46.1%)	7 (77.7%)	0.13
Angiotensin converting enzyme inhibitors	22 (62.8%)	15 (57.6%)	7 (77.7%)	0.43
Aspirin	36 (42.8%)	10 (38.4%)	5 (55.5%)	0.45
Statins	12 (34.2%)	8 (30.7%)	4 (44.4%)	0.68
Calcium channels blockers	10 (28.5%)	7 (26.9%)	3 (33.3%)	0.69

*NYHA* New York Heart Association classification of heart failure

No electrolyte imbalance was registered during the 6-month follow-up in any of the groups.

The baseline serum testosterone level was similar in the two groups (2.12 ± 0.14 in group A versus 2.09 ± 0.20 in group B, *p* = 0.62). It decreased significantly after 6 months of treatment (*p* < 0.001) (0.11 ± 0.14 in group A versus 0.07 ± 0.09 in group B, *p* = 0.45).

We analyzed and compared the variation of ECG parameters between M0 and M1 in group A versus B (Table 2). In group B at M0 QTc, QTd, max Tpe/QT, mean Tpe, max Tpe, Tped had statistically significant higher values compared to group A, but without significant variation at M1. In group A, there was a statistically significant prolongation of QTc, QTd, mean Tpe/QT, max Tpe/QT, max Tpe, Tped in M1 versus M0 and a significantly higher “delta” of these parameters than in group

**Table 2:** Variations of ECG parameters between visits in the studied groups; group A: degarelix in association with enzalutamide; group B = degarelix in monotherapy

	Group A (*n* = 26; 74.3%)			Group B (*n* = 9; 25.7%)			A vs B *M*0 *p*	A vs B *M*1 *p*	Delta Group A	Delta Group B	*p*
	M0	M1	p	M0	M1	p					
QTc (ms)	437.5 ± 40.7	470.1 ± 39.0	0.0007	490.0 ± 32.2	486.9 ± 49.2	0.82	0.001	0.30	32.6 ± 43.6	− 3.1 ± 40.9	0.03
QTd (ms)	75.3 ± 43.2	95.0 ± 33.1	0.03	111.1 ± 53.2	112.2 ± 37.3	0.94	0.05	0.20	19.6 ± 43.6	1.1 ± 47.8	0.28
Mean Tpe/QT	0.19 ± 0.04	0.21 ± 0.04	0.01	0.22 ± 0.03	0.21 ± 0.04	0.22	0.06	0.97	0.016 ± 0.034	− 0.009 ± 0.020	0.03
Max Tpe/QT	0.23 ± 0.04	0.27 ± 0.05	0.0001	0.27 ± 0.05	0.26 ± 0.05	0.43	0.02	0.67	0.034 ± 0.039	− 0.012 ± 0.045	0.004
Mean Tpe (ms)	77.0 ± 14.8	80.1 ± 20.8	0.39	94.5 ± 15.3	91.0 ± 24.0	0.53	0.005	0.20	3.1 ± 18.3	− 3.4 ± 15.9	0.34
Max Tpe (ms)	95.3 ± 17.1	115.7 ± 25.8	0.0004	123.7 ± 26.7	119.3 ± 29.4	0.66	0.0008	0.72	20.3 ± 25.7	− 4.3 ± 28.9	0.02
Tped (ms)	36.4 ± 15.6	52.5 ± 19.7	0.002	52.4 ± 19.7	51.9 ± 23.1	0.94	0.02	0.95	16.0 ± 24.3	− 0.6 ± 28.7	0.09
QTp (ms)	329.8 ± 29.3	329.2 ± 22.6	0.93	340.1 ± 16.5	347.9 ± 30.2	0.40	0.32	0.05	− 0.5 ± 32.7	7.8 ± 26.5	0.49
JTp (ms)	217.2 ± 31.2	207.7 ± 30.5	0.20	208.7 ± 30.7	224.8 ± 47.3	0.12	0.48	0.21	− 9.5 ± 16.1	16.1 ± 28.7	0.07
JTe (ms)	263.4 ± 35.5	271.5 ± 39.6	0.31	289.5 ± 41.0	313.5 ± 66.4	0.10	0.07	0.02	8.0 ± 39.8	23.9 ± 39.2	0.30
QRS (ms)	112.3 ± 20.1	112.7 ± 12.0	0.89	130.0 ± 27.4	123.5 ± 23.7	0.19	0.04	0.08	0.4 ± 18.4	− 6.4 ± 13.5	0.31
PR (ms)	158.0 ± 26.5	155.4 ± 18.6	0.67	203.1 ± 34.4	199.1 ± 34.6	0.37	0.0002	0.00003	− 2.6 ± 31.2	− 4.0 ± 12.8	0.89

B (Fig. 3).

**Fig. 3:**
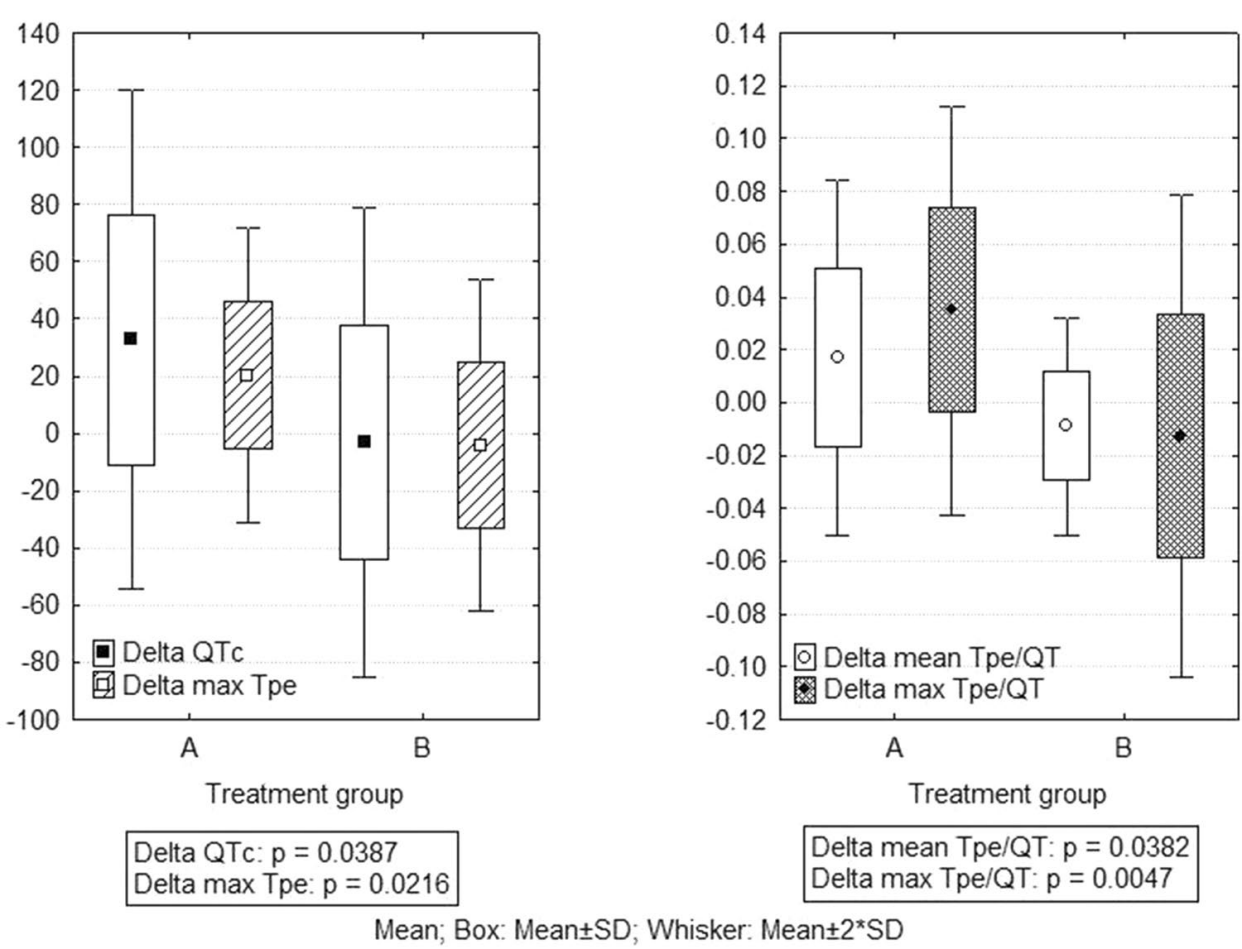
Comparison of the amount of QTc and Tpe/QT ratio variation between treatment groups: group A = degarelix in association with enzalutamide; group B = degarelix in monotherapy

**Fig. 4:**
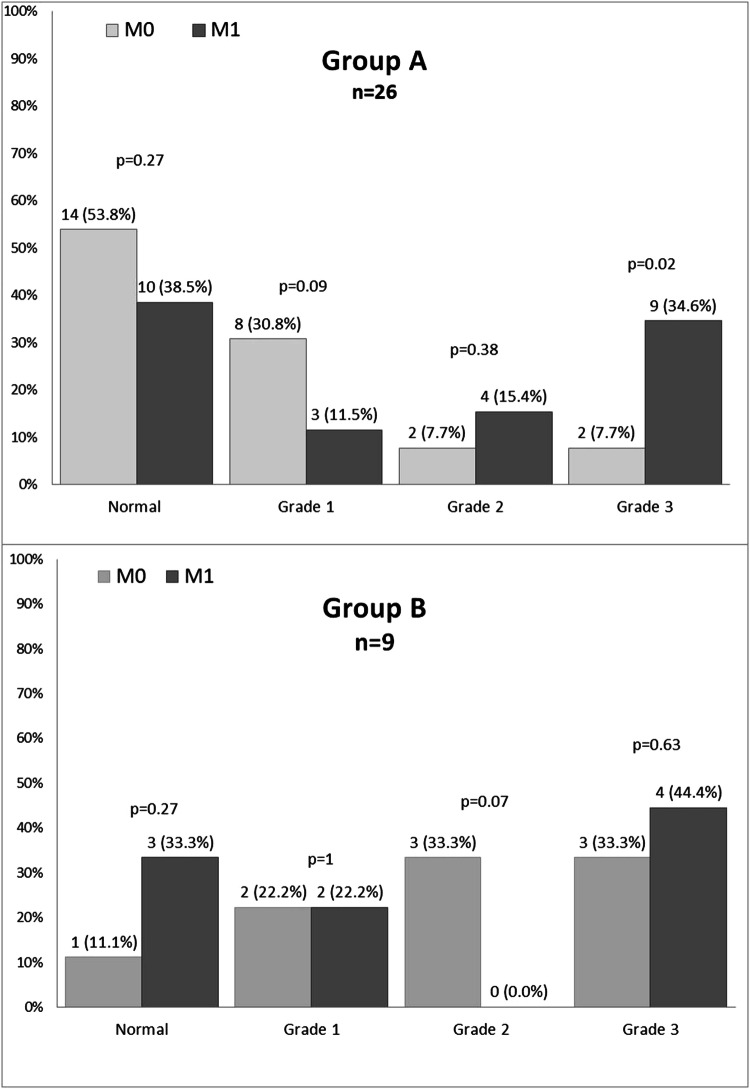
CTCAE grades of QTc prolongation in the studied groups

With respect to CTCAE grading of QTc interval prolongation, we observed an aggravation of the grades in 14 cases, with similar proportions between groups: 11(42%) patients in A and in 3 (33%) patients in B, *p* = 0.47. The distribution of CTCAE QTc interval prolongation grades between the studied groups at both visits is presented in Fig. 4. We observed a significant increase in the proportion of grade 3 QTc prolongation in group A between visits: 2 (7.7%) at M0 versus 9 (34.6%) at M1, *p* = 0.02. No CTCAE grade 4 QTc interval prolongation has been observed in neither of the groups at any of the visits.

At the same time, 6 patients in A, but none in B had more than 60 ms prolongation of QTc interval in M1 versus M0. Demographic characteristics did not differ between patients with or without a ≥ 60 ms QTc interval prolongation (*p* = NS): mean age 67.5 ± 5.5 years, history of arterial hypertension in 4 (66.6%), stable coronary disease in 4 (66.6%), diabetes mellitus in 2 (33.3%), no history of OMI.

**Fig. 5:**
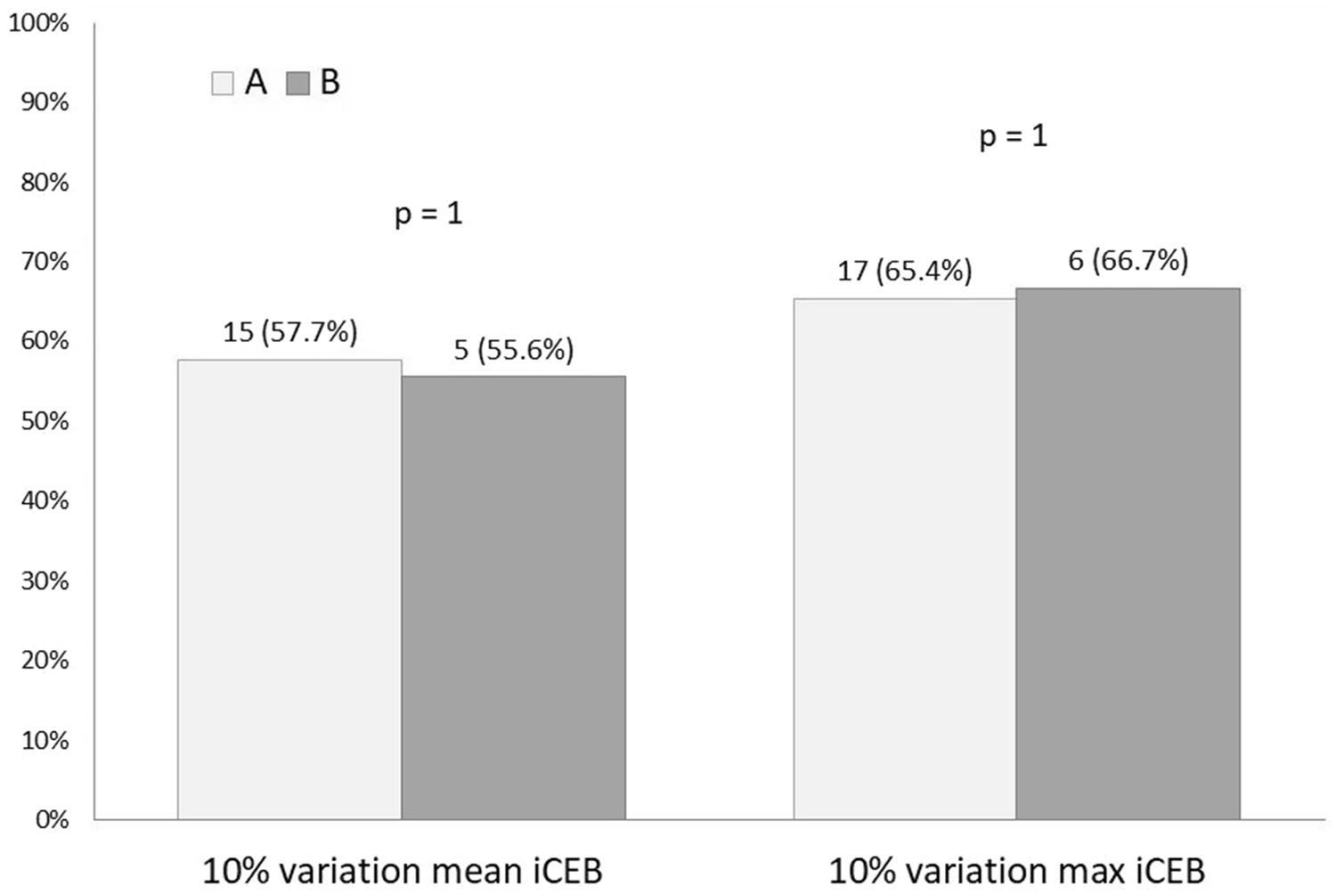
Proportion of patients with 10% iCEB variation in the studied groups between visits

Analyzing data on iCEB, we observed that the proportion of patients with a 10% variation between visits was similar in the two groups (Fig. 5). 20 (77%) patients in group A and 6 (66%) patients in group B had a variation of iCEB (mean iCEB, max iCEB or both) more than 10% (either increase or decrease) between M0 and M1, equally distributed between groups (*p* = 0.66). Their demographic data did not differ from patients without 10% iCEB variation (p = NS): mean age was 70 ± 7 years, history of hypertension was present in 20 (76.9%) patients, stable coronary disease in 15 (57.6%) patients, diabetes in 6 (23%) patients, 4 (15.3%) patients had OMI. In addition, the variations of the other ECG parameters of repolarization were similar between patients with and without 10% iCEB change between Mo and M1 (Table 3).

**Table 3:** The amount of variation “delta” of ECG parameters in relation to 10% iCEB variation

ECG parameter	With 10% iCEB variation (*n* = 26; 72.3%)	Without 10% iCEB variation (*n* = 9; 25.7%)	*P*
Delta QTc (ms)	24.4 ± 45.5	20.5 ± 46.8	0.82
Delta QTd (ms)	18.7 ± 46.9	3.8 ± 38.1	0.39
Delta mean Tpe/QT	0.01 ± 0.03	− 0.01 ± 0.02	0.22
Delta max Tpe/QT	0.02 ± 0.04	0.003 ± 0.05	0.13
Delta mean Tpe (ms)	2.5 ± 18.3	− 1.8 ± 16.4	0.53
Delta max Tpe (ms)	15.9 ± 29.6	8.3 ± 24.8	0.49
Delta Tped (ms)	10.8 ± 26.3	14.1 ± 26.8	0.74
Delta QTp (ms)	− 1.8 ± 30.7	11.4 ± 31.6	0.27
Delta JTp (ms)	− 5.0 ± 39.8	3.3 ± 27.3	0.56
Delta JTe (ms)	13.8 ± 42.1	7.1 ± 33.2	0.66

Mean duration of QRS complex was 112.3 ± 20.1 ms at M0 and 112.7 ± 12.0 ms at M1 in group A (p = 0.89) and 130 ± 27.4 ms and 123.5 ± 23.7 ms, respectively, in group B (*p* = 0.19) (Table 2). QRS complex duration was significantly longer in group B versus group A at baseline (*p* = 0.04). The higher mean value of QRS duration in group B was not associated with bundle branch block QRS pattern and was interpreted as nonspecific intraventricular conduction prolongation, 44.4% patients in group B having OMI.

PR interval duration was 158.0 ± 26.5 ms at M0 and 155 ± 18.6 at M1 (p = 0.67) in group A and 203.1 ± 34.4 ms and 199.1 ± 34.6 ms, respectively, in group B (*p* = 0.37) (Table 2). PR interval duration was significantly longer in group B versus group A at both visits (*p* < 0.001), but not reaching the first-degree atrioventricular block duration criterion.

With respect to the occurrence of VPBs during 24-h Holter monitoring, there were 5 (20%) patients in whom the severity of VPBs aggravated from non-complex to complex between visits. These patients were equally distributed according to treatment, 2 (12.5%) in group A and 3 (33.3%) in group B (*p* = 0.31). The aggravation of VPBs did not associate neither with the presence of specific cardiovascular risk factors or comorbidities nor with the treatment received. At the same time, VPBs evolved from complex in M0 to non-complex in M1 in 3 other patients. No sustained ventricular arrhythmia was registered in any of the groups during the 6-month follow-up.

The raw data are publicly available in Mendeley Data repository [12].

## Discussions

Many studies assess the occurrence of cardiovascular adverse events in patients with prostate cancer on androgen deprivation therapy [13–17], but data regarding the risk of sudden cardiac death in these patients are inconclusive. An analysis of the Surveillance, Epidemiology and End Results Medicare database which includes 73,196 patients with locoregional prostate cancer showed that the use of gonadotropin-releasing hormone receptor agonists was associated with a higher risk of coronary heart disease, sudden cardiac death, and myocardial infarction compared to orchiectomy [17, 18].

A study that included 1015 patients with prostate cancer treated with local therapy with or without androgen deprivation therapy, with a 3.8-year follow-up, found an increased risk of sudden cardiac death after 1–4 months of therapy [14], however, these results were not confirmed by other studies [13].

At the same time, there are many studies that describe a link between serum testosterone level and QT interval on ECG [19–23]. It is well known that testosterone decreases the duration of QT intervals [13, 21, 23]. The mechanism seems to be the decrease in the L-type calcium channel current and increase of several K currents including rapidly activating delayed rectifier current (*I*_k r_), slowly activating delayed rectifier current (*I*_ks_) and inward rectifier current (*I*_k1_) [3, 19, 21, 23, 24]. After orchiectomy, the QT interval becomes longer than in healthy age-matched male subjects [19]. In addition, a case series with men with long QTc and torsade de pointes reported the association between the prolongation of QTc and hypogonadism, either central or peripheric [5].

With respect to the mechanisms of the pathological changes of ECG repolarization parameters induced by androgen deprivation therapy, data are confusing. Drugs that prolong QT interval slow the rapid delayed rectifier potassium current (*I*_kr_) depending on the hERG gene and acting in the phase 3 of cardiac action potential [25]. On the other hand, experimental studies showed that degarelix had no effect on the hERG gene and membrane K channels [26]. In fact, the prolongation of the QT interval may be due to the hypogonadism induced by the drug [27]. However, O’Farrell et al. [28] studied 41,362 patients with prostate cancer treated by androgen deprivation therapy or orchiectomy in comparison with 187,785 men without prostate cancer and found that the risk of cardiovascular disease was increased by 21% in androgen deprivation therapy patients and by 16% in orchiectomy group compared to the men without prostate cancer. The risk was higher in patients with a previous history of cardiovascular disease.

There are few data regarding cardiac side effects of enzalutamide, an androgen receptor inhibitor. The product label information mentions the prolongation of QT interval on ECG particularly in patients receiving other drugs that have this effect on QT interval or who already have long QT interval. A meta-analysis involving 7 studies with 8660 patients showed that only hypertension was associated with enzalutamide [29]. On the other hand, data from the international pharmacovigilance database VigiBase showed that enzalutamide is associated with more sudden deaths than other antiandrogenic drugs [30]. Experimental studies confirm the prolongation of QT interval induced by enzalutamide [27], but apparently without clinical importance. In the PROSPER study (a phase 3, randomized, double-blind, placebo-controlled study of enzalutamide in men with nonmetastatic castration-resistant prostate cancer) involving 1401 patients without clinically significant cardiovascular disease, the most frequently reported adverse effects were hypertension and fatigue [31]. However, recently published data demonstrated a relation between enzalutamide and abnormalities induced in the *I*_Kr_ cardiac repolarization current. An experimental study [30] using cardiomyocytes derived from induced pluripotent stem cells demonstrated that enzalutamide in acute or chronic administration inhibits delayed rectifier potassium current, prolongs action potential duration, produces afterdepolarizations and triggered activity and in chronic administration, enhances late sodium current. On the ECG tracings, androgen deprivation therapy induces QTc interval prolongation, QRS duration reduction, T wave flattening, or notching [30].

In our study, a significant prolongation of QTc, max Tpe, mean Tpe/QT, max Tpe/QT at M1 versus M0 occurred in patients in group A receiving degarelix associated with enzalutamide, but not in those in group B treated with degarelix in monotherapy. Also, the amount of variation “delta” for these ECG parameters was significantly greater in group A than B. The mean “delta” in group A was 32.6 ± 43.6 ms. In the literature, the mean prolongation of QTc reported with androgen deprivation therapy is 10–20 ms [32]. We interpreted that patients in group A had a more pronounced QTc prolongation in comparison with the data reported in the literature due to the association of two classes of antiandrogens, whose cardiac effects may enhance each other. In addition, in our study, 6 patients in group A had a QTc interval prolongation of more than 60 ms. The significant variation of the ECG indices of repolarization in group A occurred regardless of their baseline value, either normal or prolonged*.* In fact, values of QTc, Tpe, Tpe/QT were higher at M0 in group B than in group A probably because of the greater prevalence of old myocardial infarction. This could represent an argument for the supposition that the effect of androgen deprivation therapy is less dependent on the baseline values of repolarization parameters, but may be related to the antiandrogen medication itself, in our case enzalutamide and degarelix. Our findings raise the question of whether the deleterious effects of combining enzalutamide and degarelix on cardiac repolarization are additive.

Gagliano-Jucá et al. [33] described the shortening of QRS complex duration in patients receiving androgen deprivation therapy. In our study, the QRS complex duration was greater in group B, probably related to the higher proportion of patients with old myocardial infarction, but in none of the groups was a significant shortening of its duration recorded.

So far there is no information regarding the evolution of PR interval in patients on androgen deprivation therapy. In our study, there was no significant variation of this interval.

In experimental studies, a 10% variation (either increase or decrease) of iCEB values from baseline showed to be a promising marker for drug-induced arrhythmic risk [7, 34]. However, data from clinical trials are scarce. In this study, the proportion of patients who met the 10% iCEB variation criterion (mean iCEB, max iCEB or both) was similar in the two groups: 20 (77%) patients in group A and 6 (66%) in group B (*p* = 0.66). Patients with 10% iCEB variation presented similar demographic characteristics and ECG parameter values compared to patients with no significant iCEB variation. Thus, in our study, there was no association between prolongation of repolarization markers reflecting arrhythmic risk and the 10% variation of iCEB.

When synthesizing our findings, we consider that it is difficult to distinguish between the effects of hypogonadism and those of androgen deprivation therapy on the repolarization parameters. In our study, the occurrence of significant prolongation of ECG repolarization parameters occurred in patients treated by degarelix in co-treatment with enzalutamide and not in patients treated with degarelix in monotherapy, despite their similar degree of hypogonadism after 6 months of treatment. This fact raises the question of whether there is an enhanced effect of this association of drugs on the occurrence of the cardiac repolarization changes.

Taking into account the findings of Piccirillo et al. [35], who studied 14 hypogonadal patients and 10 age-matched controls and found no difference in Tpe, we can assume that the significant prolongation of Tpe and Tpe/QT in our study group A reflects the effect of the combination of degarelix with enzalutamide, not of the hypogonadism itself.

Another finding of our study is related to the timing of repolarization changes: they begin in the first 6 months of therapy. Salem et al. [30], analyzing data from the international pharmacovigilance database VigiBase regarding men with acquired long QT syndrome, torsade de pointes or sudden death associated with antiandrogenic therapy, found that sudden cardiac death occurred after minimum 0.25 days of administration of antiandrogen deprivation therapy with a median time of 92 days [30].

We observed an aggravation of VPBs from non-complex to complex in 5 (20%) patients who performed the Holter ECG study, which has not been associated with the presence of any cardiovascular risk factors or with the treatment received. During the 6 months of treatment, no sustained ventricular arrhythmia was registered. Thus in the short term, disturbances of repolarization parameters in patients with hypogonadism induced by degarelix in co-treatment with enzalutamide do not seem to be associated with the occurrence of severe ventricular arrhythmias.

Our study and its results had several limitations. Firstly, although the design was that of a prospective observational study, patients had a relatively short follow-up period. Secondly, the two groups were small. Thirdly, we did not analyze other ECG repolarization changes shown to be drug induced like T wave notching. At this point, we cannot recommend which ECG repolarization parameter is the most useful for the arrhythmic risk stratification in patients treated by antiandrogen therapy, and while adding max Tpe, max Tpe/QT, Tped parameters to classical QTc analysis seems promising, further research is necessary. Although the alteration of the ECG repolarization parameters occurred during the first 6 months of treatment, we did not observe any clinical consequences during this period. Consequently, we cannot provide an analysis of the long-term cardiovascular effects of degarelix associated or not with enzalutamide in patients with advanced prostate cancer and further research is needed.

In conclusion, after a 6-month treatment period, patients with advanced prostate cancer and hypogonadism treated with degarelix associated with enzalutamide had significant prolongations of QTc, QTd, maxTpe, meanTpe/QT, maxTpe/QT, Tped compared to those treated with degarelix in monotherapy. Despite the difficulty of differentiating between the effects of hypogonadism and those of antiandrogenic drugs on the ECG parameters of arrhythmic risk, we consider that the combination of degarelix and enzalutamide worsens the repolarization changes. Adding Tpe, Tpe/QT, and Tped to QTc analysis might be useful for arrhythmic risk assessment. No serious arrhythmic consequences were recorded during the first 6 months of treatment in any of the two groups.
